# Phytotoxicity of *Schiekia timida* Seed Extracts, a Mixture of Phenylphenalenones

**DOI:** 10.3390/molecules26144197

**Published:** 2021-07-10

**Authors:** Fernanda Maria Marins Ocampos, Ana Julia Borim de Souza, Guilherme Medeiros Antar, Felipe Christoff Wouters, Luiz Alberto Colnago

**Affiliations:** 1 Embrapa Instrumentação, São Carlos CEP 13560-970, SP, Brazil; 2 Faculdade de Ciências, Universidade Estadual Paulista “Júlio de Mesquita Filho” (UNESP), Bauru CEP 17033-360, SP, Brazil; anajuliaborim@gmail.com; 3 Instituto de Biociências, Departamento de Botânica, Universidade de São Paulo (USP), Butantã, São Paulo CEP 05508-090, SP, Brazil; guilherme.antar@gmail.com; 4 Departamento de Química, Universidade Federal de São Carlos (UFSCAR), São Carlos CEP 13565-905, SP, Brazil; fcwouters@ufscar.br

**Keywords:** phenylphenalenones, *Schiekia timida*, phytotoxicity, NMR, UPLC-HRMS

## Abstract

Phenylphenalenones, metabolites found in *Schiekia timida* (Haemodoraceae), are a class of specialized metabolites with many biological activities, being phytoalexins in banana plants. In the constant search to solve the problem of glyphosate and to avoid resistance to commercial herbicides, this work aimed to investigate the phytotoxic effect of the methanolic extract of *S. timida* seeds. The chemical composition of the seed extract was directly investigated by NMR and UPLC-QToF MS and the pre- and post-emergence phytotoxic effect on a eudicotyledonous model (*Lactuca sativa*) and a monocotyledonous model (*Allium cepa*) was evaluated through germination and seedling growth tests. Three concentrations of the extract (0.25, 0.50, and 1.00 mg/mL) were prepared, and four replicates for each of them were analyzed. Three major phenylphenalenones were identified by NMR spectroscopy: 4-hydroxy-anigorufone, methoxyanigorufone, and anigorufone, two of those reported for the first time in *S. timida*. The presence of seven other phenylphenalenones was suggested by the LC-MS analyses. The phenylphenalenone mixture did not affect the germination rate, but impaired radicle and hypocotyl growth on both models. The effect in the monocotyledonous model was statistically similar to glyphosate in the lowest concentration (0.25 mg/mL). Therefore, although more research on this topic is required to probe this first report, this investigation suggests for the first time that phenylphenalenone compounds may be post-emergence herbicides.

## 1. Introduction

Competition between crops and weeds, sharing the same environment, causes financial losses to farmers. In order to avoid this competition, herbicides are the most used, but not ideal, approach to control weed spreading [1,2]. Their use enabled increasing crop production and optimizing prices for the consumer [3]. However, resistance to commercial herbicides is increasing and, to solve this crisis, new chemical skeletons with different modes of action are required [4]. There are two major types of herbicides based on the mode of action: systemic or translocated and contact herbicides [5]. The first acts by interfering with the biochemical processes required for normal growth and development, taking several days to eliminate the plant (e.g., glyphosate). On the other hand, contact herbicides (e.g., clove oil) damage the plant structures where the herbicide touches, being more active in young plants than in older ones, which can regenerate. According to Singh et al., 2020, from the mode of action point of view, herbicides can be cell membrane disruptors, inhibitors of amino acid biosynthesis, lipid biosynthesis inhibitors, photosynthesis inhibitors, plant growth regulators, nitrogen metabolism inhibitors, pigment inhibitors, or seedling growth inhibitors [5]. The most widely used herbicide is the organophosphorus glyphosate, a systemic post-emergence herbicide, which affects the biosynthesis of phenylalanine, tyrosine, and tryptophan. Plants produce these aromatic amino acids by the shikimate pathway, and for this reason, glyphosate is a post-emergence herbicide. Its use increased in the ’90s due to the introduction of transgenic glyphosate-resistant crops [6], which are resistant to this compound. The wide use of glyphosate gave rise to naturally resistant weeds by many mechanisms, diminishing its effectiveness, and demanding new solutions to integrated weed management [7]. To illustrate this problem, Vidotto et al. (2020) demonstrated the development of resistance in the weed *Echinochloa* spp. to which the same herbicide was applied repeatedly over time [8]. According to Beckie et al., 2019, in the last 30 years, herbicides with new modes of action have not been commercialized. Among the reasons for the lack of novelty is the extensive use of glyphosate, a post-emergence herbicide, in transgenic resistant crops, for instance, soybean, maize, and cotton [9]. Trends in this topic involve lowering weed seed banks and reduced dependence on glyphosate and claim the need of application of the Commoner’s five principles of ecology to achieve success in herbicide resistance management [2,9,10]. In summary, the best methodology is to combine proactive (e.g., herbicide mixtures and new active ingredients) with reactive strategies (e.g., mechanical control) [11]. An interesting approach to solve this problem is the discovery of plant-derived active compounds, one of the less explored sources of novel molecular target sites for herbicides [12]. In this way, new natural chemical scaffolds may be a way to accomplish new solutions in integrated weed management.

From the chemical ecology point of view, living organisms communicate through molecules. This communication happens intending to perpetuate species and, therefore, compounds are produced with innumerable objectives, such as attracting pollinators in plants and fighting pathogens [13]. One type of communication between plants is allelopathy, in which specialized metabolites of a given plant are released to the environment and affect the development of other surrounding species. This is a natural process that occurs in the plant habitat. By contrast, in phytotoxicity investigations metabolites are extracted *in vitro* by methods such as maceration in organic solvents [14]. According to Reigosa et al. (1999), defense specialized metabolites may have a secondary role to help to perpetuate the species by affecting competitors growing nearby [15]. Hence, the diversity of plant specialized metabolites, such as the phenylphenalenones investigated in this work, represent a valuable source of new herbicide chemical scaffolds.

Phenylphenalenones are phytoalexins in resistant cultivars of banana plants (*Musa* sp.), although they are also found in Haemodoraceae, Pontederiaceae, and Strelitziaceae [16]. Phenylphenalenones are biosynthesized by the phenylpropanoids pathway that after condensation with malonyl-CoA give rise to diarylheptanoids which suffer intramolecular cyclization [17,18,19]. The antifungal activity that protects the resistant banana cultivars against diseases such as black Sigatoka and Panama disease drove the attention to this class of metabolites. Since then, other biological activities, for instance, antioxidant [20], antiplasmodial [21], and nematicide [22] have been demonstrated. Ocampos et al., 2017, in a tissue-specific occurrence investigation of rhizomes, stems, leaves, flowers, and fruits, confirmed that *Schiekia* Meisn., an Haemodoraceae previously monospecific genus endemic of South America, is a source of phenylphenalenones [23]. At that time, the species investigated was treated as *Schiekia orinocensis* (Kunth) Meisn., but, after a recent taxonomic revision [24], it is now recognized as *Schiekia timida* M.Pell., E.J.Hickman, Rhian J.Sm. & Hopper, due to its different ecological preferences and morphology. In Brazil, this plant composes a drink used in religious ceremonies of the Krahô tribe. *S. timida*, refers to the “shy” flowers that slightly open mostly during the rainy season of the seasonally flooded grasslands of South America’s north region [24].

NMR and LC-MS the most widely used techniques for the investigation of natural products, with complementary characteristics. NMR is considered a non-destructive, reliable, and reproducible tool. By analyzing a combination of obtained spectra and correlation maps, one can obtain chemical structures unambiguously [25,26]. It is useful even in mixtures, giving the relative concentration of all the compounds in the sample, and when the correct parameters are used, it can also give the absolute concentration of each compound in a given sample. These qualities of NMR spectroscopy make it possible to not only identify but also quantify samples in one single analysis, giving the perfect idea of all the major compounds in the sample [27,28,29,30]. However, despite increasing efforts to improve their sensitivity, NMR analyses still face challenges in the investigation of minor components of mixtures. Such limitations can be overcome by MS analyses, which show overall high sensitivity and are easily coupled with separation by UPLC, allowing for the comprehensive profiling of complex mixtures. Additionally, high-resolution MS provides exact mass measurements that can be used to suggest molecular formulas and further confirm those structures elucidated by NMR [31]. When used in combination, NMR and LC-MS provide complementary, comprehensive, quantitative, and precise information on the composition of complex mixtures such as plant extracts [32].

Thus, this work aimed to investigate the phenylphenalenone composition of *S. timida* seed extract by NMR and LC-MS as well as to evaluate its phytotoxic effect on two model species representing monocotyledonous (*Allium cepa* L.) and eudicotyledonous (*Lactuca sativa* L.) plants, pursuing new possibilities of new compounds with herbicide activity.

## 2. Results

*S. timida* dry fruits were collected from a habitat located at the municipality of Novo Acordo, at Tocantins state, Brazil (Geographical coordinates: 10°15′26.8″ S, 47°21′22.3″ W, 271 m). Seeds were separated from the other fruit parts and extracted directly with deuterated solvents. The extract was directly analyzed by NMR, from which three major phenylphenalenones were identified. Further analysis by UPLC-QToF MS confirmed the molecular formulas of the three major compounds identified by NMR and suggested the presence of 7 other minor phenylphenalenones.

### 2.1. Identification and Annotation of Phenylphenalenones

**Figure 1:**
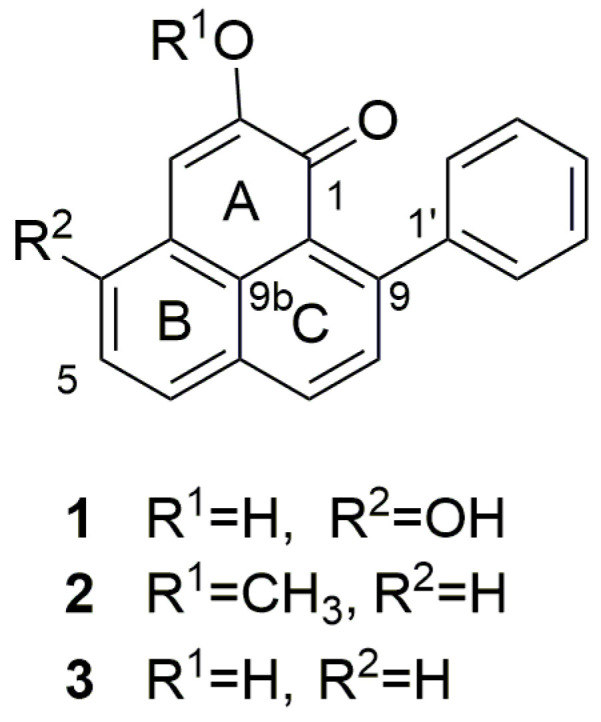
Chemical structures of phenylphenalenones found in *Schiekia timida* seeds extract.

The chemical investigation on the methanolic extract of *S. timida* seeds revealed three major compounds (Figure 1) identified as 4-hydroxy-anigorufone (**1**) [33], methoxyanigorufone (**2**) [34], and anigorufone (**3**) [34]. NMR data is available in Table S2 and Figures S2–S5). The extract also presented other minor phenylphenalenone characteristic NMR signals that could not be identified by NMR due to the low concentrations in the extract.

**Figure 2:**
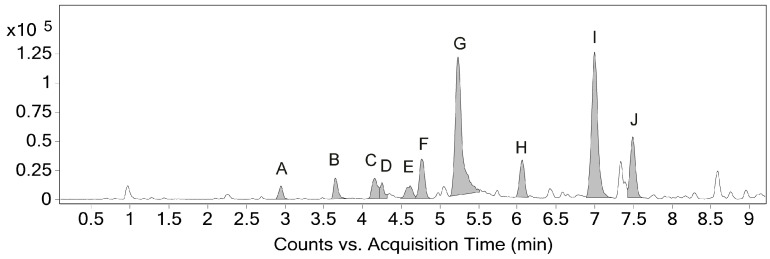
Base peak chromatogram (0–9 min) of *Schiekia timida* seed methanolic extract; 10 phenylphenalenone peaks (A–J) are highlighted with molecular formulas suggested by UPLC-QToF MS in positive ESI mode.

Chromatographic peaks (Figure 2) from the base peak chromatogram in positive ESI mode were manually inspected and 10 peaks (A–J, Table 1) representing phenylphenalenones were selected based on molecular formulas suggested by high-resolution MS spectra. The three major peaks, G, I, and J, presented the expected [M+H]^+^ signals for the structures identified by NMR for compounds **1**, **2,** and **3**, respectively. These major compounds were further analyzed by MS^2^ experiments in positive mode, revealing fragmentation patterns that supported the tentative identification of the other 7 minor phenylphenalenone derivatives. Mass spectra in ESI negative mode were used to aid in the identification of corresponding [M+H]^+^ ions of low abundance in positive mode but were not used for the suggestion of molecular formulas, given their general poor ionization efficiency, low abundance, and large error for most peaks. All positive and negative (when available) MS and MS^2^ spectra are presented in the Supplementary Information (Figures S8–S17).

The most intense peaks of the chromatogram presented [M+H]^+^ signals with *m*/*z* compatible with the major chemical structures identified by NMR. The proportion between chromatographic peak areas gave a ratio of 44% to 41% to 15% for peaks G, I, and J, respectively. Accordingly, the relative quantification performed by NMR indicates that the integrals for those compounds (Figure S6) have a ratio of 53% to 33% to 14% (compounds **1**, **2**, and **3**, respectively), when they are normalized to a 100%.

### 2.2. Seed Germination and Phytotoxicity

**Figure 3:**
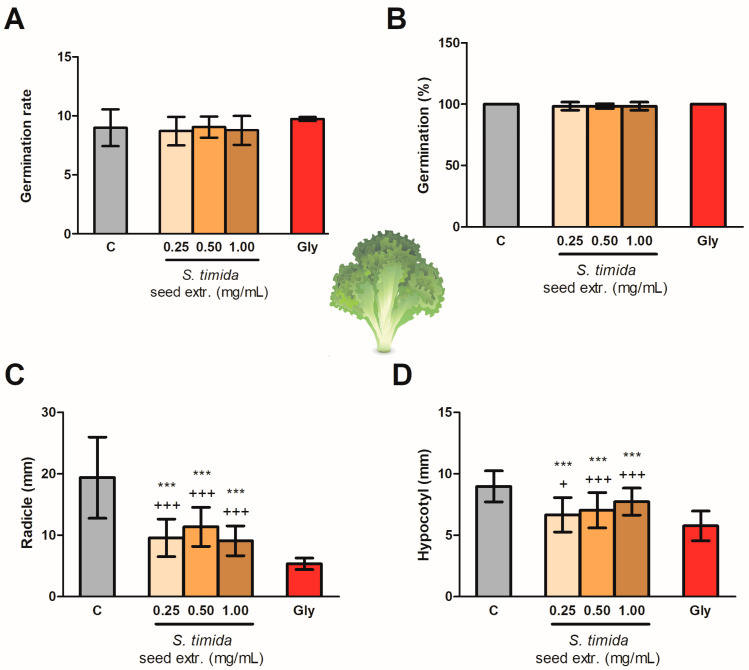
Effect of *Schiekia timida* seed extracts (0.25, 0.50, and 1.00 mg/mL) on *Lactuca sativa* germination rate (**A**, ANOVA *p* = 0.7155), germination percentage (**B**, ANOVA *p* = 0.6402), and radicle (**C**, ANOVA *p* < 0.0001) and hypocotyl (**D**, ANOVA *p* < 0.0001) growth in comparison to a C (negative control) and Gly (glyphosate), expressed by mean ± SD. *** Significant difference (*p* < 0.001) in comparison to C, + Significant difference (*p* < 0.05) in comparison to Gly, and +++ Significant difference (*p* < 0.001) in comparison to Gly, by Tukey’s Multiple Comparison Test.

Pre-emergence test results of *L. sativa* seeds exposed to different concentrations of *S. timida* extracts are shown in Figure 3. The seed germination was not affected by the extracts or glyphosate (Gly), as shown in Figure 3A,B. On the other hand, the extracts in the concentrations of 0.25, 0.50, and 1.00 mg/mL altered the hypocotyl (6.65 ± 1.41, 7.03 ± 1.44, and 7.72 ± 1.11 mm, respectively) and radicle (9.55 ± 3.05, 11.35 ± 3.19, and 9.06 ± 2.43 mm, respectively) lengths, being statistically different from both control groups (Figure 3C,D). Thus, an intermediate effect between water (C) and Gly on the seedling growth was observed.

**Figure 4:**
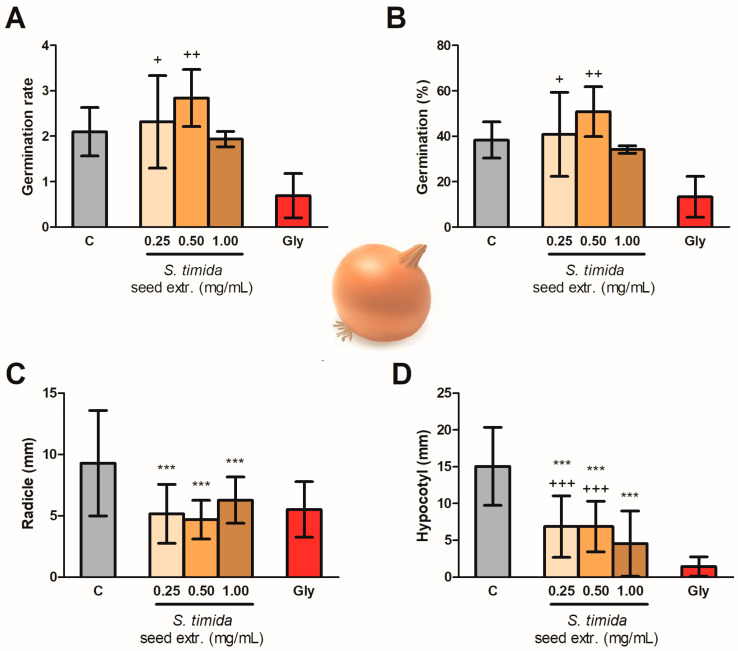
Effect of *Schiekia timida* seed extracts (0.25, 0.50, and 1.00 mg/mL) on *Allium cepa* germination rate (**A**, ANOVA *p* = 0.0033), germination percentage (**B**, ANOVA *p* = 0.0036), and radicle (**C**, ANOVA *p* < 0.0001) and hypocotyl (**D**, ANOVA *p* < 0.0001) growth in comparison to a C (negative control) and Gly (glyphosate) expressed by mean and SD. *** Significant difference (*p* < 0.001) in comparison to C, + Significant difference (*p* < 0.05) in comparison to Gly, ++ Significant difference (*p* < 0.005) in comparison to Gly and +++ Significant difference (*p* < 0.001) in comparison to Gly, by Tukey’s Multiple Comparison Test.

A similar pattern was found for the germination of the monocotyledonous *A. cepa*. Seed extracts of *S. timida* presented an effect statistically similar to the negative control group, showing that the germination was not affected by the treatments. Glyphosate, however, did slow the germination (Figure 4A,B). Figure 4C shows the radicle growth of onion seedlings, which was affected already by the lowest concentration extract (*p* < 0.001). The effect of the extracts of 0.25 and 0.50 mg/mL on the hypocotyl growth was statistically different from both C and Gly (Figure 4D). Therefore, these two concentrations presented an intermediate effect in the development of the hypocotyl. The highest concentration (1.00 mg/mL) of *S. timida* extracts inhibited the hypocotyl and radicle growth with a non-significant difference from Gly (Figure 4C,D).

## 3. Discussion

The methanolic extract of *S. timida* seeds is composed mostly of a mixture of three phenylphenalenones with unmodified C_19_ skeletons (Figure 1 and Table S2). This is the first time that compounds **1** and **3** were found in *S. timida*. Moreover, this also the first report of the phytotoxic activity of phenylphenalenones. Ocampos et al. (2017), in a tissue-specific occurrence investigation including rhizomes, stems, leaves, flowers, and fruits, isolated methoxyanigorufone (**2**) only from the fruits of *S. timida* [23]. This suggests that in *S. timida* C_19_ phenylphenalenones skeletons may be exclusive compounds in fruits and seeds or are present in very low concentrations in other parts of the plant.

Compounds **1**, **2**, and **3** are 9-phenylphenalenones found in Haemodoraceae plants and phytoalexins in *Musa* sp [35]. Biosynthetically, anigorufone (**3**) is formed by the symmetrical incorporation of two molecules of cinnamic acid and *p*-coumaric acid [36,37]. Full NMR assignments (Table S2) of the ^1^H and ^13^C NMR spectra, as well as 1-D and 2-D NMR spectra and correlation maps, are presented in the supplementary information file.

The molecular formula of **3** (*R_t_* 7.49, Figure 2) was determined as C_19_H_12_O_2_ (Table 1, Figure S17), based on the [M+H]^+^ peak at *m/z* 273.0917 in the HRMS spectrum (calcd. 273.0910). The ^1^H NMR spectrum showed multiplet signals at δ 7.31 (2H, H-2′/6′), δ 7.39 (2H, H-3′/5′), and 7.34 (1H, H-4′), which are assignable to the phenyl ring protons; an AB spin system of H-8 (δ 7.52) and H-7 (δ 8.25); another spin system of H-4 (δ 7.71), H-5 (δ 7.58) and H-6 (δ 7.94); and a singlet of H-3 (δ 7.06). HMBC correlations (Figure S4) of H-8 with C-1′ (δ 144.8), C-6a (δ 133.0) and C-9a (δ 126.1.2), and of H-7 with C-6 (δ 130.6), C-9 (δ 149.8) and C-9b (δ 126.3) established the structure of rings C and D. The HMBC H-3 correlations with C-1 (δ 181.8), C-2 (δ 151.9), C-4 (δ 131.1) and C-9b (δ 126.3), established the structures of rings A and B, including the carbonyl at C-1. The HMBC cross-signals of H-4 with C-3 (δ 114.4), C-6 (δ 130.6), and C-9b completed the structural assignment of the skeleton of compound **3**.

Compound **2** (*R_t_* 7.00, Figure 2) was determined as C_20_H_14_O_22_ (Table 1, Figure S16), based on the [M+H]^+^ peak at *m*/*z* 287.1077 in the HRMS spectrum (calcd. 287.1067). The NMR signals showed a similar structure to compound **3**, except that an HMBC (Figure S4) showed a different correlation between H-3 and C-2 (δ 154.2) correlating with an *O*-methyl signal (3 H, δ 3.98) assigned the methoxy group to C-2.

Compound **1** (*R_t_* 5.23, Figure 2), presented a different NMR profile to ring B. The HMBC (Figure S4) correlation of H-6 with C-4 presented a higher chemical shift (δ 159.4) than on the other compounds, compatible with the presence of a hydroxyl group attached to the carbon. The HRMS spectrum (Table 1, Figure S15) confirmed the chemical structure based on the [M+H]^+^ peak at *m*/*z* 289.0868 in the HRMS spectrum (calcd. 289.0859), compatible with the molecular formula C_19_H_12_O_3_.

MS^2^ analyses of compounds **1** and **3** confirm the fragmentation of both [M+H]^+^ precursor ions generating the main signals observed in their MS spectra, including a loss of H_2_O (theor. *m*/*z* 18.0106), possibly followed by a loss of CO (theor. *m*/*z* 27.9949). A similar MS profile is observed for peaks A, B, and C, suggesting they share structural features with **1** and **3**. In contrast, MS^2^ analysis of compound **2** confirms a loss of CH_4_ (theor. *m*/*z* 16.0313) from the [M+H]^+^ precursor ion, which differs from the other two identified compounds and can be associated with the presence of a methoxy group. Likewise, a CH_4_ loss is also suggested in the MS spectra of peaks D, E, F, and H, indicating they are also methoxy derivatives. Accordingly, the ^1^H NMR spectrum of the methanolic extract presents four minor methoxyl singlets at δ3.91, δ3.95, δ3.96, and δ3.98. Their HMBC correlations, however, indicate that they are attached to carbons of lower chemical shifts (δ145.2 for the first three and δ146.1 for the last one).

Interestingly, compound **1** displayed an intense peak in negative ESI mode, while compounds **2** and **3** did not seem to ionize efficiently (Figure S7). This might be attributed to the 4-hydroxyl group in compound **1** since the presence of a 2-hydroxyl group in compound **3** was not sufficient to yield a significant [M–H]^−^ ion. Similar to compound **1**, peaks **A**, **C**, **D,** and **F** also appeared in both positive and negative modes, suggesting that these derivatives may have hydroxyl groups in positions other than C-2.

From a biological activity point of view, phenylphenalenone compounds are constitutively biosynthesized by Haemodoraceae plants, although they are known to be phytoalexins in resistant cultivars of banana plants (*Musa* sp.) against pathogens such as *Mycosphaerella fijiensis* and *Fusarium oxysporum* [16,38,39]. Compound **1** presents radical scavenging capacity with antioxidant activity due to a hydrogen atom transfer (HAT) mechanism of action, being the hydroxyl group at C-4 the most relevant feature for this activity [20]. Compound **3** is also a natural product of *Strelitzia reginae* and has antinematode, leishmanicidal, and antimicrobial activity in the concentration of 1 mg/mL, presenting activity against *Bacillus subtilis*, methicillin-resistant *Staphylococcus aureus*, *Escherichia coli*, *Pseudomonas aeruginosa*, *Sporobolomyces salmonicolor*, *Mycobacterium vaccae*, *Candida albicans,* and *Penicillium notatum* [40,41,42,43,44]. Therefore, as these chemical scaffolds present potential biological activity, other bioactivities have been investigated [20,21,42,45,46,47] and the phytotoxic activity of phenylphenalenones is reported for the first time in the present work.

Thus, in order to investigate the phytotoxic activity of this class of compounds, two model species representing monocotyledonous (*A. cepa*) and eudicotyledonous (*L. sativa*) plants were used to evaluate the phytotoxicity of phenylphenalenones [2,48]. An interesting approach would be to separate the compounds by preparative chromatography and to investigate the biological activity of the isolated compounds. This would make it possible to investigate and compare each chemical structure with the obtained activity and give a better idea of the most active compounds. However, considering the low availability of botanical material to obtain enough isolated compounds to perform a statistically significant biological assay and the structural similarity between the compounds, in this case, the best explorative approach was to use crude extracts on the biological assays.

The phenylphenalenones from *S. timida* seed extract did not affect seed germination on both models tested (lettuce and onion) and, therefore, this class of compounds seems to affect the post-emergence of plants as demonstrated in Figure 3 and Figure 4. The results are also presented in Table S1, which is available in the supplementary information file. The extracts were active after seeds germinated, having an intermediate effect between control and glyphosate groups on the eudicotyledonous model. The monocotyledonous model presented a better response with activity statistically similar to glyphosate (1.69 mg/mL) for the inhibition of radicle growth in all the tested concentrations, with statistically significant activity (*p* < 0.001) on the lowest tested seed extract concentration (0.25 mg/mL), as the radicle, an indispensable structure is to plant development and establishment, was affected. Moreover, the effect was not dose-dependent, within the tested range, with no statistical difference in the radicle length between the three concentrations. The concentration of *S. timida* methanolic seed extract required for the observed activity was 6.76 lower than the concentration used for the control glyphosate. The mechanism of inhibition observed in this assay is still not elucidated.

**Figure 5:**
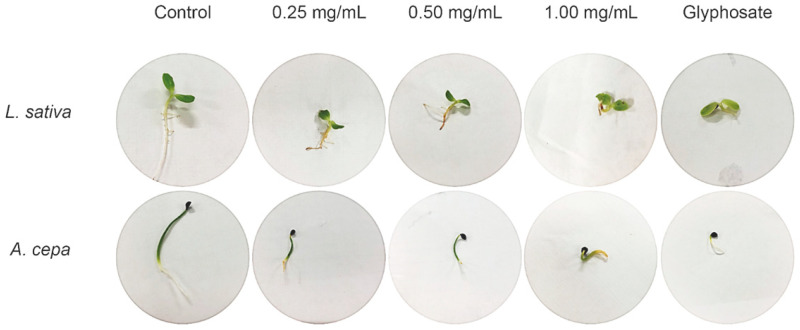
Seedlings of *Lactuca sativa* and *Allium cepa* after 7 days of germination, showing the effect of each concentration treatment (0.25, 0.50, and 1.00 mg/mL) in comparison to control (water) and glyphosate.

Phenylphenalenones are orange to red pigments. Therefore, it was possible to visualize that these pigments were absorbed by plant structures by the orange color of the radicle in treated seedlings (Figure 5).

The phytotoxic activity to the models tested in this work was more severe to the monocotyledonous *A. cepa*. By contrast, phenylphenalenones are biosynthesized by monocotyledonous plants (Musaceae, Haemodoraceae, Strelitziaceae, and Pontederiaceae). This highlights the necessity of elucidating the mechanism of resistance of the phenylphenalenones-producing plants on further investigations. Other issues to be investigated are whether crops are tolerant to phenylphenalenones as well as the best protocols for application. Nevertheless, it is known that the efficacy in delaying weed resistance is higher when combining multiple effective herbicide sites of action in a single application than when rotating herbicides [49,50]. And in this way, a complex mixture such as a plant extract may be an alternative. However, this approach alone is not sufficient and a more diverse approach is required, combining mechanical and chemical intervention [10].

*S. timida* grows in the field by vegetative propagation and only a few other species seem to grow in the surroundings. The present results, together with field observations, suggest that an allelopathic activity might occur. However, further investigations are required to test whether such phytotoxic activities are relevant in the natural context and whether phenylphenalenones play an ecological role in *S. timida* biology as allelopathic secondary metabolites.

The commercial use of phenylphenalenone scaffolds would only be practical without the need of isolating them from natural sources, as they are normally biosynthesized in extremely low amounts in plants. Efforts to synthesize these compounds have been made [47,51,52,53,54,55,56,57], but the high concentration of phenylphenalenones found in methanolic extracts of *S. timida* seeds raises the possibility of using this species as an alternative source of phenylphenalenones scaffolds.

## 4. Materials and Methods

### 4.1. General Procedures

^1^H NMR, ^1^H-^1^H COSY, ^1^H-^13^C HSQC, and ^1^H-^13^C HMBC spectra were acquired on a Bruker AVANCE III HD 600 NMR spectrometers (Bruker, Karlsruhe, Germany) operating at 14.1 T, observing ^1^H and ^13^C at 600.13 and 150.90 MHz, respectively. Spectrometers were equipped with a 5-mm multinuclear detection probe with a z-gradient (Bruker). NMR spectra were measured at 300 K in MeOH-*d*_4_ or acetone-*d*_6_ and D_2_O (1:1, *v*/*v*). In the ^1^H NMR spectra, the noesypr1d pulse sequence was used to suppress the residual water signal. Each ^1^H-NMR spectrum consisted of 128 scans on a spectral width of ~20 ppm, 0.73 Hz/point, acquisition time (AQ) = 1.36 s, relaxation delay (RD) = 2.0 s, 90° pulse width (PW) = 11.4 µs. One-bond (HSQC) and long-range (HMBC) ^1^H-^13^C NMR correlation experiments were optimized for average coupling constants ^1^*J*_(H,C)_ and ^LR^*J*_(H,C)_ of 140 and 8 Hz, respectively. Spectra were processed using TopSpin 3.5 software (Bruker). All ^1^H and ^13^C NMR chemical shifts were observed in ppm related to TMS signal at 0.00 ppm as an internal reference and exponential line broadening of 0.3 Hz were applied. After Fourier transformation, spectra were manually phased, and baselines were corrected. Metabolites were assigned based on the chemical shifts, signal multiplicities, and integrals always in comparison to literature. Relative quantification of the major compounds was addressed by integrating isolated signals of ^1^H NMR spectrum of *S. timida* methanolic seed extract. The integrals corresponding to one hydrogen of each molecule were normalized to 100% and the contribution of each was calculated. The chosen signals were the doublets H-6 at δ7.85 for compound **1**, and H-4 at δ7.78 and δ7.71 for compounds **2** and **3**, respectively.

LC-MS analyses were performed on an Agilent 1260 Infinity II UPLC connected to an Agilent 6545 Q-ToF. Chromatographic analyses used an Agilent Zorbax Eclipse Plus C18 (2.1 × 50 mm, 1.8 μm) column, kept at 35 °C, using an injection volume of 2 μL, a flow rate of 400 μL min^−1^ and a binary MS-grade solvent system of water (solvent A) and acetonitrile (solvent B), both containing 0.1% (*v*/*v*) formic acid. Sample volumes were loaded onto the column and eluted by using the following gradient: linear increase from 30% B to 80% B within 9 min − 100% B constant for 3 min − with an equilibration time set at 30% B for 3 min. Total ion spectra were collected in the mass range of *m*/*z* 100 to 1000 in positive and negative ion modes at a rate of 5 spectra per second. The drying gas temperature and flow were 325 °C and 12 L/min, respectively. The temperature and flow of the sheath gas were 350 °C and 11 L/min, respectively. The nebulizer gas pressure was 25 psi, and the skimmer, fragmentor, nozzle, and capillary voltage were 65 V, 150 V, 1000 V, and 3500 V, respectively. LC-MS data were analyzed using Agilent MassHunter Qualitative Analysis Navigator (Version B.08.00, Build 8.0.8208.0) software.

MS^2^ experiments were performed in automatic mode, AutoMS2, selecting the 5 most intense MS ions per cycle. Precursor ions were fragmented using argon as a collision gas with a fixed collision energy of 15 V.

### 4.2. Plant Material

*S. timida* seeds were collected in June 2019 in Novo Acordo, Tocantins, Brazil. Geographical coordinates: 10°15′26.8″ S, 47°21′22.3″ W, 271 m. An exemplar of the species was collected and identified by one of us, G.M. Antar, and a voucher specimen (G.M. Antar 2717—SPF 248229) has been deposited at the Herbarium SPF of the University of São Paulo (USP).

### 4.3. Extraction and Identification of Natural Compounds

Seeds (3.0 g) were grounded in liquid N_2_ and extracted with 40 mL of methanol in ultrasound for 20 min. The extraction procedure was repeated three times with the same plant material. The extract was dried on the rotatory evaporator to give 129.7 mg (4.3% yield). This extract was used for phytotoxicity assays and chemical characterization through LC-MS analyses. For the NMR analysis, powdered plant material (100 mg) was transferred to a 2 mL microtube. The extraction was performed with 1.5 mL of deuterated methanol (CD_3_OD) containing 0.1% *w*/*w* of tetramethylsilane (TMS). After 20 min ultrasonication, the mixture was centrifuged at 6585.6 g for 20 min, and 600 μL of the supernatant was transferred to a 5 mm NMR tube.

### 4.4. Phytotoxicity Bioassay

The methodology was adapted from Macías et al., 2000 [58]. *S. timida* seed extract was resuspended in ultrapure water in three concentrations (0.25, 0.50, and 1.00 mg/mL). Eudicotyledonous (*L. sativa* L.) and monocotyledonous (*A. cepa* L.) seeds were acquired in the local market and the bioassay was set using Petri dishes (140 × 15 mm) with one sheet of Whatman No. 1 filter paper as support. Thirty seeds and 5 mL of extract were added to each plate. Water was used as a negative control and an aqueous solution of glyphosate (10 mmol/L or 1.69 mg/mL) as a positive control. Four biological replicates to each experimental group were prepared. Seeds were incubated in a growth chamber at 20 °C, 80% moisture, and 14/10 h of light/dark cycle.

#### 4.4.1. Germination Test

The protrusion of 2 mm and geotropic curvature of the radicle was used as germination criteria. The seeds that showed false germination by soaking were not accounted for in the results. The germination was determined by daily counting the number of germinated seeds for 7 days. The indices used to evaluate germination and seedling growth were calculated as described by Maguire, 1962 [59].

#### 4.4.2. Seedling Growth Test

After the 7 days of germination, the radicle and hypocotyl lengths of 10 seedlings of each plate (n = 40 for each experimental group) were measured in mm.

### 4.5. Statistical Analysis

The statistical treatment was performed using GraphPad Prism 5, v 5.01 software after the normality (Shapiro-Wilks) was performed using the Origin 9.3 software. As the data presented normal distribution, the results were analyzed using 1-way ANOVA followed by Tukey’s Multiple Comparison Test (*p* < 0.001).

## 5. Conclusions

This manuscript explored the phytotoxicity of phenylphenalenones for the first time. The chemical composition of *S. timida* seeds, never investigated before, was accessed and three phenylphenalenones were identified as the major compounds in the methanolic extract, which contains at least seven other annotated minor phenylphenalenones. The mixture of phenylphenalenones from *S. timida* methanolic seeds extracts was tested in three different concentrations aiming to explore potential herbicide sources. The extract activity was statistically similar to glyphosate, already in the lowest concentration (0.25 mg/mL) for the monocotyledonous model. The eudicotyledonous model was impaired as well, although at a lower level. The extract did not interfere with the seed germination rate, and, therefore, it may be considered a post-emergence herbicide, as is glyphosate. This report is the first step to highlight the possibility of using phenylphenalenones scaffolds to solve the herbicide resistance problem. Further studies on other crops are, however, needed in order to explore the potential of these compounds and extracts in agriculture.

## Figures and Tables

**Table 1:** Retention times, experimental and theoretical masses [M+H]^+^ for the phenylphenalenones present in the methanolic extract of *Schiekia timida*, indicating the data obtained for compounds **1**, **2**, and **3**.

Compound	Peak	RT (min)	Formula (M)	*m*/*z* Theoretical	*m*/*z* exp.	Error (ppm)
	A	2.95	C_19_H_14_O_4_	307.0965	307.0968	0.98
	B	3.65	C_19_H_12_O_4_	305.0808	305.0814	1.97
	C	4.16	C_19_H_12_O_4_	305.0808	305.0814	1.97
	D	4.25	C_19_H_12_O_4_	305.0808	305.0817	2.95
	E	4.62	C_20_H_12_O_4_	319.0965	319.0970	1.57
	F	4.75	C_20_H_14_O_3_	303.1016	303.1022	1.98
**1**	G	5.23	C_19_H_12_O_3_	289.0859	289.0869	3.46
	H	6.06	C_19_H_12_O_4_	305.0808	305.0816	2.62
**2**	I	7.00	C_20_H_14_O_2_	287.1067	287.1077	3.48
**3**	J	7.49	C_19_H_12_O_2_	273.0910	273.0919	3.30

## Data Availability

The data presented in this study are available on request from the corresponding author.

## Supplementary Materials

The following are available online, Figure S1. Seedlings of *L. sativa* and *A. cepa* after 7 days of germination, Figure S2. ^1^HNMR spectrum of *Schiekia timida* seeds extract (MeOH-d_4_, 600 MHz, 300 K), Figure S3. Expansion (8.40–6.90 ppm) of HSQC correlation map of *Schiekia timida* seeds extract (MeOH-d_4_, 600 MHz, 300 K), Figure S4. Expansion (8.40–6.90 ppm) of HMBC correlation map of *Schiekia timida* seeds extract (MeOH-*d*_4_, 600 MHz, 300 K), Figure S5. Expansion (8.40–6.90 ppm) of COSY correlation map of *Schiekia timida* seeds extract (MeOH-d_4_, 600 MHz, 300 K), Figure S6. Expansion (8.90–6.68 ppm) of ^1^H NMR spectrum of *Schiekia timida* seeds extract (MeOH-d_4_, 600 MHz, 300 K), Figure S7. Base peak chromatogram (0–9 min) of *S. timida* seed extract; UPLC-QToF MS in negative ESI mode, Figure S8. Mass spectrum of peak A (2.95 min) in positive and negative ESI modes, Figure S9. Mass spectrum of peak B (3.65 min) in positive ESI mode, Figure S10. Mass spectra of peak C (4.16 min) in positive and negative ESI modes, Figure S11. Mass spectra of peak D (4.25 min) in positive and negative ESI modes, Figure S12. Mass spectrum of peak E (4.62 min) in positive ESI mode, Figure S13. Mass spectra of peak F (4.75 min) in positive and negative ESI modes, Figure S14. Mass spectra of peak G (5.23 min), compound **1**, in positive and negative ESI modes, Figure S15. Mass spectrum of peak H (6.06 min) in positive ESI mode, Figure S16. Mass spectra of peak I (7.00 min), compound **2**, in positive and negative ESI modes, Figure S17. Mass spectra of peak J (7.49 min), compound **3**, in positive and negative ESI modes, Table S1. Phytotoxicity of *S. orinocencis* seeds extracts on different concentrations, Table S2 ^1^H, ^13^C and HMBC NMR chemical shifts (ppm) and correlations of compounds **1**, **2**, and **3**.

## Sample Availability

Samples of *S. timida* extracts are available from the authors.
