# Supplementary material for: Phytotoxicity of Schiekia timida Seed Extracts, a Mixture of Phenylphenalenones
Source: Molecules. 2021 Jul 10;26(14):4197. doi: 10.3390/molecules26144197 (PMC8304753; doi:10.3390/molecules26144197)
Supplement: Supplementary file 1 [file molecules-26-04197-s001.zip › molecules-1274809-supplementary.pdf]

molecules

MDPI

Article

Phytotoxicity of *Schiekia timida* Seed Extracts, a Mixture of Phenylphenalenones

Fernanda Maria Marins Ocampos [1,*], Ana Julia Borim de Souza [2], Guilherme Medeiros Antar [3], Felipe Christoff Wouters [4] and Luiz Alberto Colnago [1,*]

[1] Embrapa Instrumentação, CEP 13560-970, São Carlos, SP, Brazil
[2] Faculdade de Ciências, Universidade Estadual Paulista "Júlio de Mesquita Filho" (UNESP), CEP 17033-360, Bauru, SP, Brazil; anajuliaborim@gmail.com
[3] Instituto de Biociências, Departamento de Botânica, Universidade de São Paulo (USP), Butantã, CEP 05508-090, São Paulo, SP, Brazil; guilherme.antar@gmail.com
[4] Departamento de Química, Universidade Federal de São Carlos (UFSCAR), CEP 13565-905, São Carlos, SP, Brazil; fcwouters@ufscar.br
* Correspondence: fmmocampos@gmail.com (F.M.M.O.); luiz.colnago@embrapa.br (L.A.C.)

Supplementary information

Figures

2 .... ***Figure S1***. *Seedlings of L. sativa and A. cepa after 7 days of germination.*
4 .... ***Figure S2.*** *^{1}HNMR spectrum of Schiekia timida seeds extract (MeOH-d$_4$, 600 MHz, 300 K).*
5 ..... ***Figure S3*** *Expansion (8.40 - 6.90 ppm) of HSQC correlation map of Schiekia timida seeds extract (MeOH-d$_4$, 600 MHz, 300 K).*
6 ... ***Figure S4.*** *Expansion (8.40 - 6.90 ppm) of HMBC correlation map of Schiekia timida seeds extract (MeOH-d$_4$, 600 MHz, 300 K).*
6 ..... ***Figure S5.*** *Expansion (8.40 - 6.90 ppm) of COSY correlation map of Schiekia timida seeds extract (MeOH-d$_4$, 600 MHz, 300 K).*
7 .... ***Figure S6.*** *Expansion (8.90- 6.68 ppm) of ^{1}H NMR spectrum of Schiekia timida seeds extract (MeOH-d$_4$, 600 MHz, 300 K).*
7 .... ***Figure S7***. *Base peak chromatogram (0 - 9 min) of S. timida seed extract; UPLC-QToF MS in negative ESI mode.*
7 .... ***Figure S8.*** *Mass spectrum of peak A (2.95 min) in positive and negative ESI modes.*
8 .... ***Figure S9.*** *Mass spectrum of peak B (3.65 min) in positive ESI mode.*
8 .... ***Figure S10.*** *Mass spectra of peak C (4.16 min) in positive and negative ESI modes.*
8 .... ***Figure S11.*** *Mass spectra of peak D (4.25 min) in positive and negative ESI modes.*
8 .... ***Figure S12.*** *Mass spectrum of peak E (4.62 min) in positive ESI mode.*
9 .... ***Figure S13.*** *Mass spectra of peak F (4.75 min) in positive and negative ESI modes.*
9 .... ***Figure S14.*** *Mass spectra of peak G (5.23 min), compound **1**, in positive and negative ESI modes.*
9 .... ***Figure S15***. *Mass spectrum of peak H (6.06 min) in positive ESI mode.*
10 .... ***Figure S16.*** *Mass spectra of peak I (7.00 min), compound **2**, in positive and negative ESI modes.*
10 .... ***Figure S17.*** *Mass spectra of peak J (7.49 min), compound **3**, in positive and negative ESI modes.*

Tables

2 .... ***Table S1.*** *Phytotoxicity of Schiekia timida seeds extracts on different concentrations.*
3 .... ***Table S2*** *^{1}H, ^{13}C and HMBC NMR chemical shifts (ppm) and correlations of compounds **1, 2,** and **3**.*

Table S1. Phytotoxicity of *Schiekia timida* seeds extracts on different concentrations (0.25, 0.50, and 1.00 mg/mL) in comparison to C (negative control, water) and Gly (positive control, glyphosate (10^{-2} M)) expressed by the mean and standard deviation (SD) of germination rate, germination percentage, radicle length (mm) and hypocotyl length (mm) of *Lactuca sativa* L. and *Allium cepa L.*

		**Germination rate**	**Germination (%)**	**Radicle**	**Hypocotyl**
	C	8.992±1.561	100.0±0.0	19.38±6.610	8.971±1.267
	0.25 mg/mL	8.707±1.215	98.33±3.333	9.550±3.055	6.650±1.406
L. sativa	0.50 mg/mL	9.046±0.895	98.33±1.925	11.35±3.190	7.027±1.443
	1.00 mg/mL	8.767±1.227	98.33±3.333	9.065±2.435	7.725±1.109
	Gly (10^{-2} M)	9.729±0.1718	100.0±0.0	5.325±0.9443	5.750±1.214
	C	2.099±0.5329	38.33±7.935	9.289±4.305	15.05±5.312
	0.25 mg/mL	2.317±1.018	40.83±18.53	5.158±2.400	6.865±4.191
A. cepa	0.50 mg/mL	2.840±0.6289	50.83±11.01	4.675±1.575	6.875±3.451
	1.00 mg/mL	1.936±0.1700	34.17±1.667	6.275±1.881	4.550±4.449
	Gly (10^{-2} M)	0.6917±0.4851	13.33±9.027	5.516±2.264	1.440±1.325

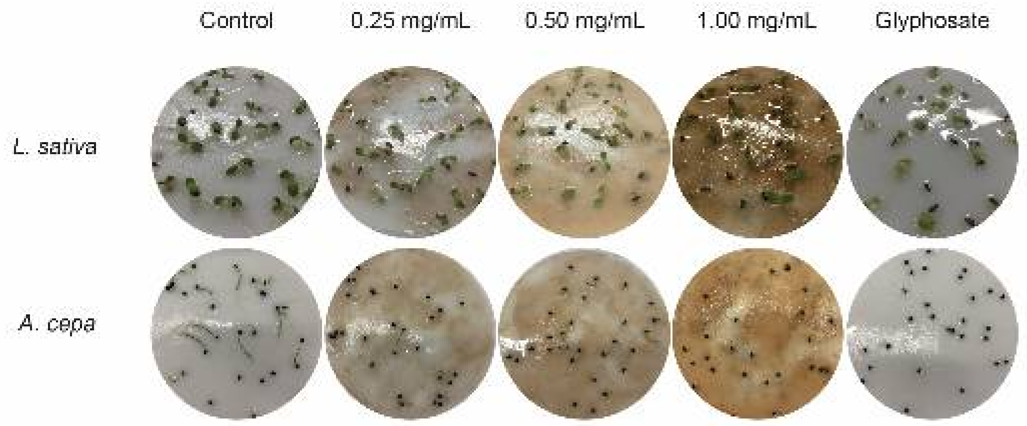

Figure S1. Seedlings of *Lactuca sativa* and *Allium cepa* after 7 days of germination

molecules

Table S2. ^{1}H, ^{13}C and HMBC NMR chemical shifts (ppm) and correlations of compounds **1**, **2,** and **3** directly identified from *Schiekia timida* seeds extracts (MeOH-d_4, 600 MHz, 300 K)

	1			2			3		
	**2,4-dihydroxy-9-phenyl-1H-phenalen-1-one RT 2.95**			**Methoxyanigorufone (2-methoxy-9-phenyl-1H-phenalen-1-one) RT 7.00**			**anigorufone (2-hydroxy-9-phenyl-1H-phenalen-1-one) RT 7.49**		
**position**	δ_C, type	δ_H, (*J* in Hz)	HMBC[a]	δ_C, type	δ_H, (*J* in Hz)	HMBC[a]	δ_C, type	δ_H, (*J* in Hz)	HMBC[a]
**1**	n.d.			181.8, C			181.8, C		
**2**	n.d.			154.2, C			151.9, C		
**3**	111.8, CH	7.36 s	4	115.5, CH	7.10 s	1, 2 , 4, 9b	114.4, CH	7.06 s	1, 2 , 4, 9b
**3a**	122.8, C			129.7, C			129.7, C		
**4**	159.4,C			131.7, CH	7.78 d (7.0)	3,6, 9b	131.1, CH	7.71 d (7.0)	3,6, 9b
**5**	119.8, CH	7.18 d (8.9)	3a, 6a	128.1, CH	7.58 dd (8.1, 7.0)	3a, 6a	128.1, CH	7.58 dd (8.1, 7.0)	3a, 6a
**6**	133.7, CH	7.85 d (8.9)	4, 7, 9b	132.6, CH	7.95 d (8.1)	4, 7, 9b	130.6, CH	7.94 d (8.1)	4, 7, 9b
**6a**	128.4, C			133.1, C			133.0, C		
**7**	135.8, CH	8.10 d (7.9)	6, 9, 9b	136.2, CH	8.24 d (8.2)	6, 9, 9b	136.2, CH	8.25 d (8.2)	6, 9, 9b
**8**	127.9, CH	7.32 (7.9)d	6a, 9a, 1′	132.5, CH	7.52 d (8.2)	6a, 9a, 1′	132.5, CH	7.52 d (8.2)	6a, 9a, 1′
**9**	150.3, C			149.8, C			149.8, C		
**9a**	124.9, C			126.1, C			126.1, C		
**9b**	127,6, C			126,3, C			126,3, C		
**1′**	144.8, C			144.8, C			144.8, C		
**2′**	129.2 CH	7.31 m	9, 4', 6'	129.2 CH	7.31 m	9, 4', 6'	129.2 CH	7.31 m	9, 4', 6'
**3′**	129.1, CH	7.39 m	1', 5'	129.1, CH	7.39 m	1', 5'	129.1, CH	7.39 m	1', 5'
**4′**	127.9, CH	7.34, m	2', 6'	127.9, CH	7.34, m	2', 6'	127.9, CH	7.34, m	2', 6'
**5′**	129.1, CH	7.39 m	1', 3'	129.1, CH	7.39 m	1', 3'	129.1, CH	7.39 m	1', 3'
**6′**	129.2 CH	7.31 m	9, 2', 4'	129.2 CH	7.31 m	9, 2', 4'	129.2 CH	7.31 m	9, 2', 4'
**OCH_3**				56.0, CH_3	3.85 s	2			

n.d. = not detected by the experiment

molecules

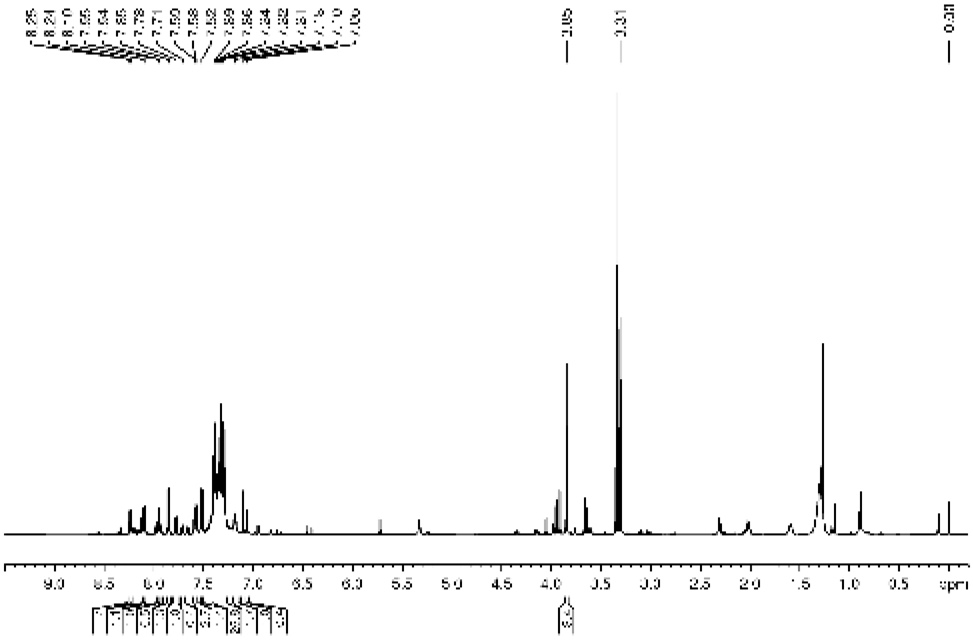

Figure S2. ^{1}HNMR spectrum of *Schiekia timida* seeds extract (MeOH-d_4, 600 MHz, 300 K), showing the chemical shifts and integrals of compounds **1**, **2,** and **3**, TMS and solvent.

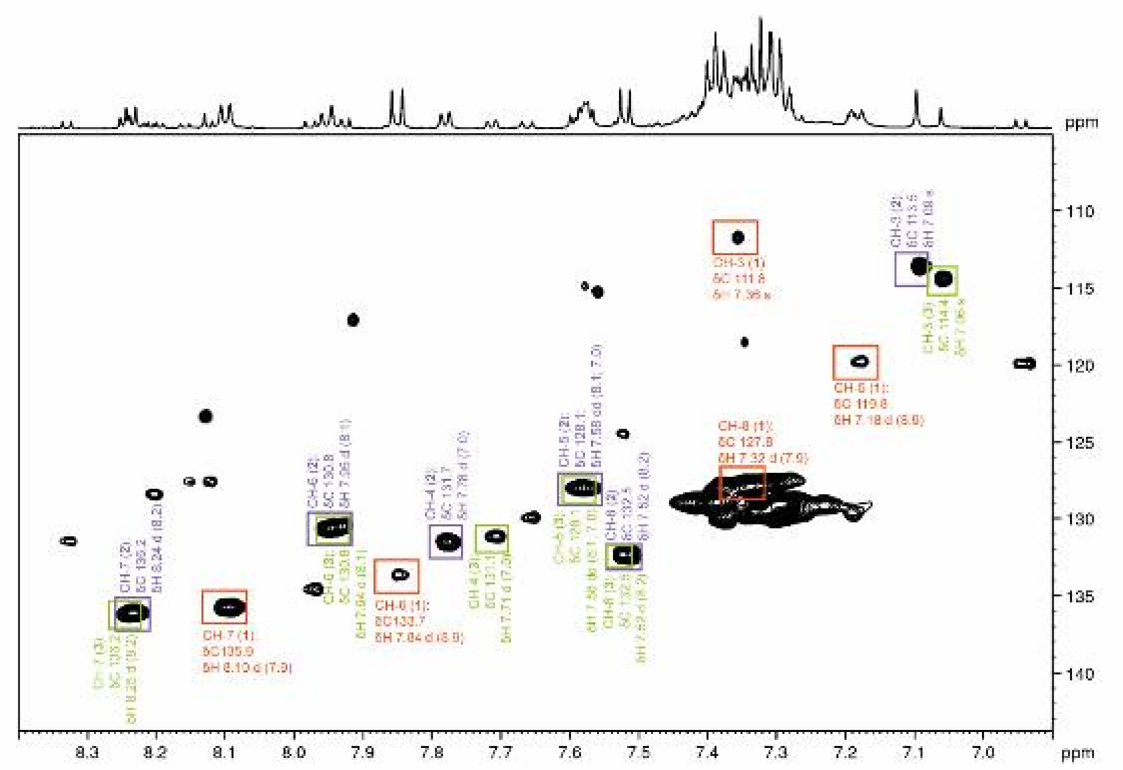

Figure S3. Expansion (8.40 - 6.90 ppm) of HSQC correlation map of *Schiekia timida* seeds extract (MeOH-d_4, 600 MHz, 300 K), showing the attributions of compounds **1**, **2,** and **3**.

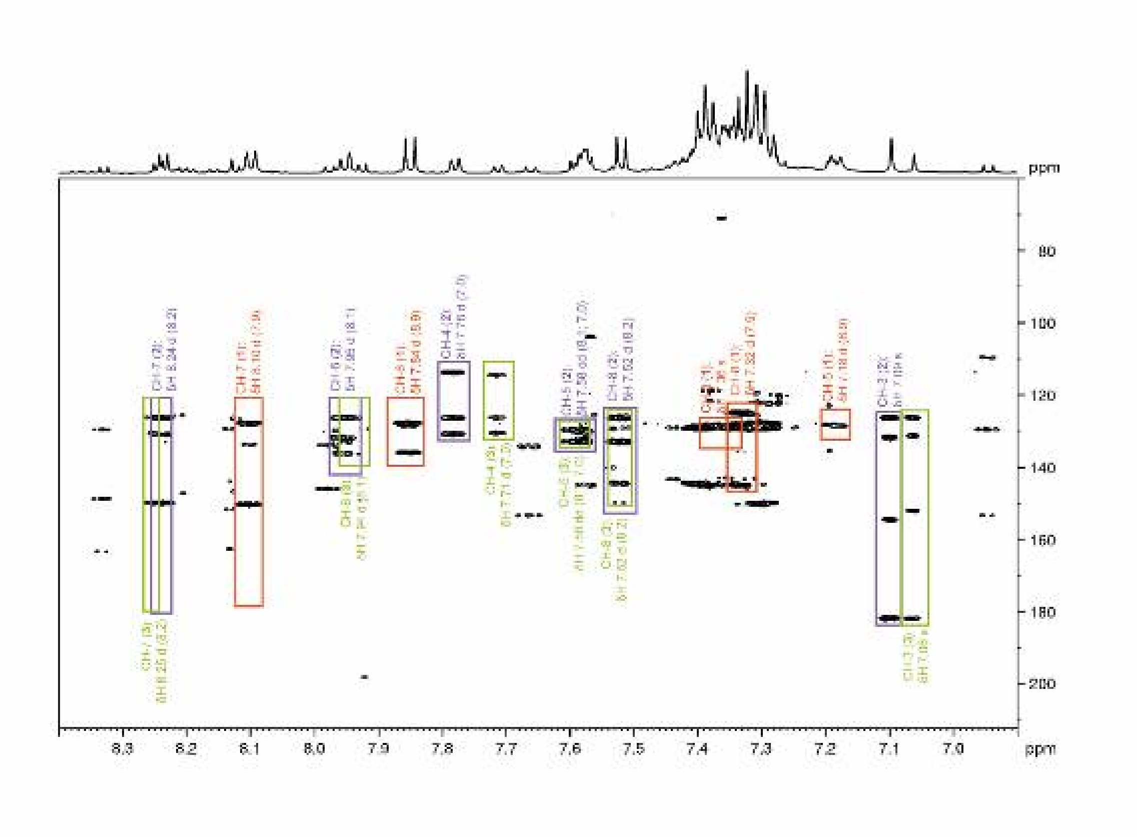

Figure S4. Expansion (8.40 - 6.90 ppm) of HMBC correlation map of *Schiekia timida* seeds extract (MeOH-d_4, 600 MHz, 300 K), showing the attributions of compounds 1, 2, and 3.

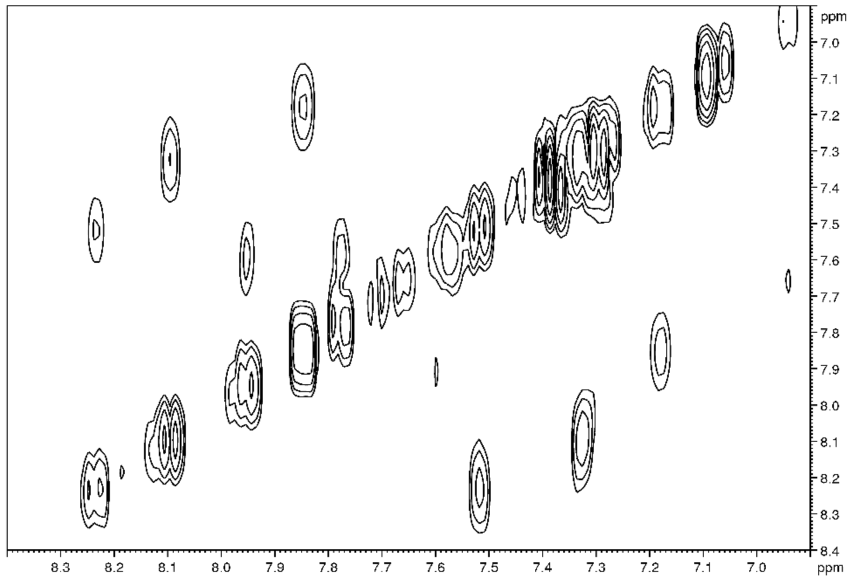

Figure S5. Expansion (8.40 - 6.90 ppm) of COSY correlation map of *Schiekia timida* seeds extract (MeOH-d_4, 600 MHz, 300 K).

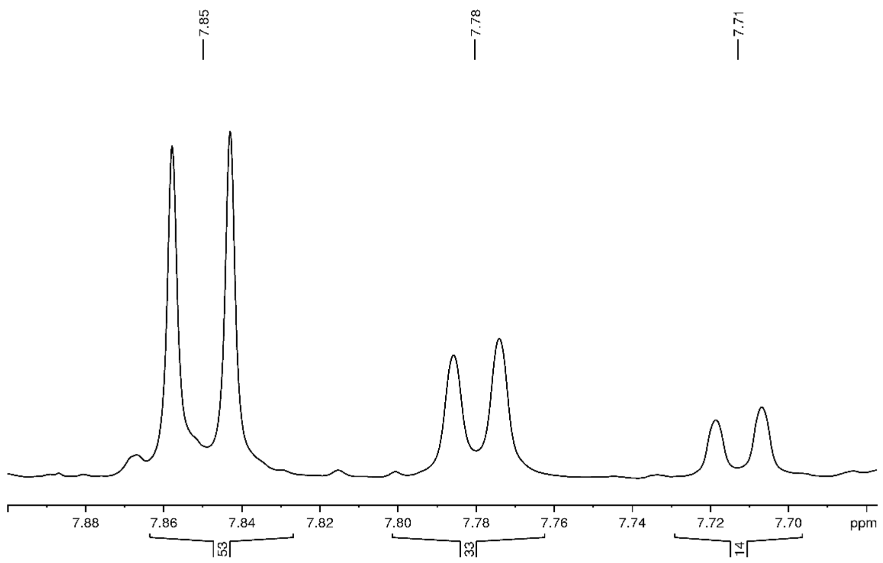

Figure S6. Expansion (8.90- 6.68 ppm) of ^{1}H NMR spectrum of *Schiekia timida* seeds extract (MeOH-d$_4$, 600 MHz, 300 K), showing the integrals normalized to 100 % of selected isolated signals of the three major compounds 1 (H-6, 1H, 7.85 ppm), 2 (H-4, 1H, 7.78 ppm), and 3 (H-4, 1H, 7.71 ppm).

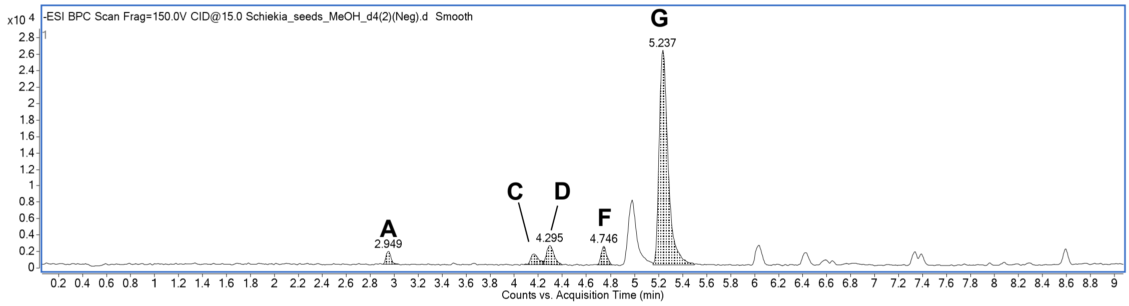

Figure S7. Base peak chromatogram (0 - 9 min) of S. timida seed extract; UPLC-QToF MS in negative ESI mode.

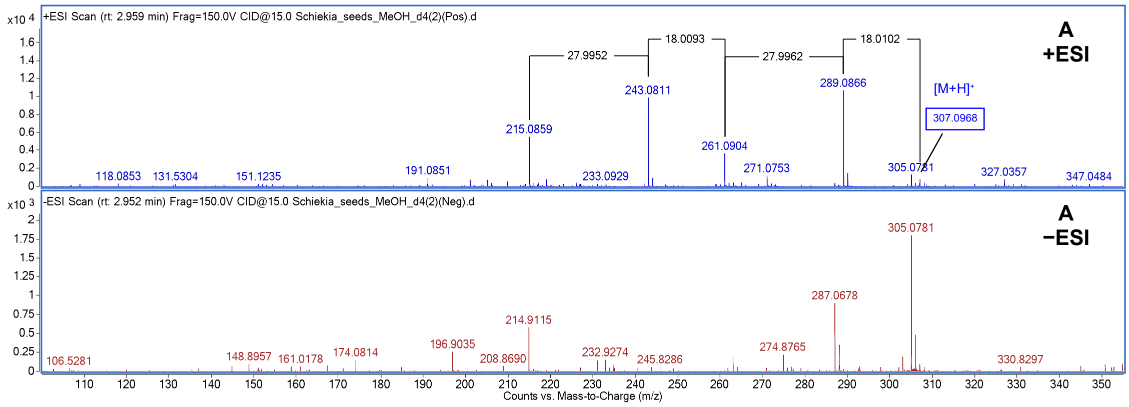

Figure S8. Mass spectrum of peak A (2.95 min) in positive and negative ESI modes.

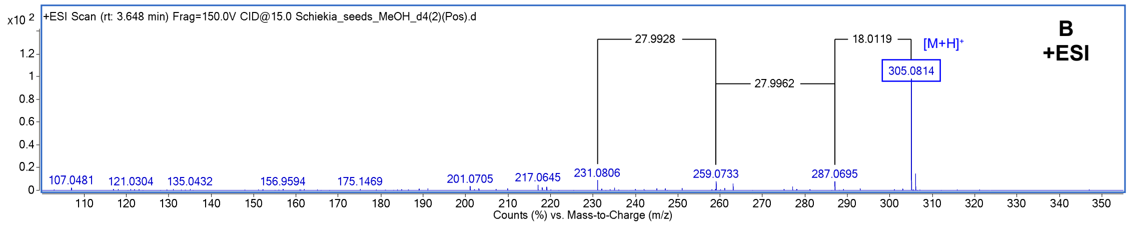

Figure S9. Mass spectrum of peak B (3.65 min) in positive ESI mode.

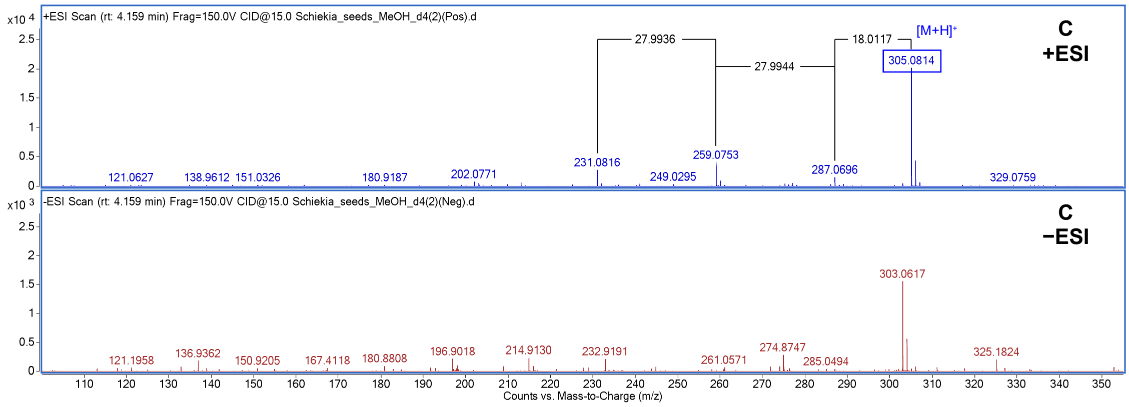

Figure S10. Mass spectra of peak C (4.16 min) in positive and negative ESI modes.

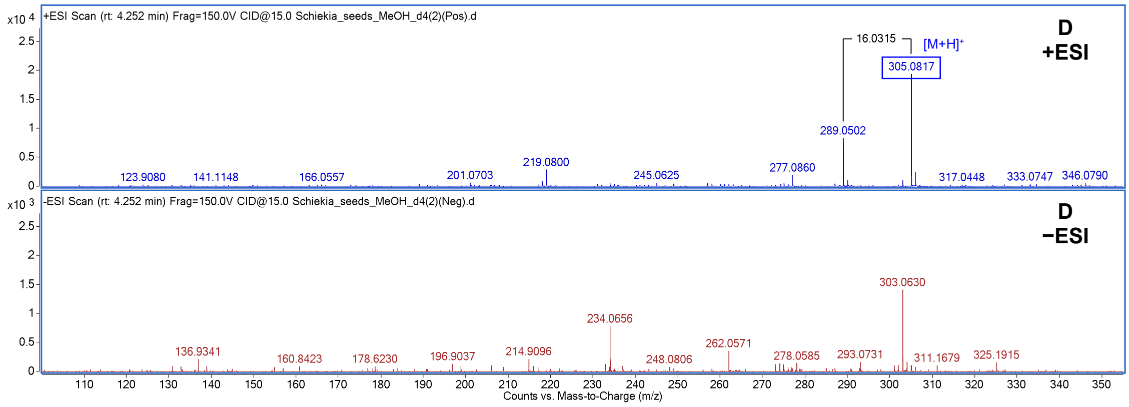

Figure S11. Mass spectra of peak D (4.25 min) in positive and negative ESI modes.

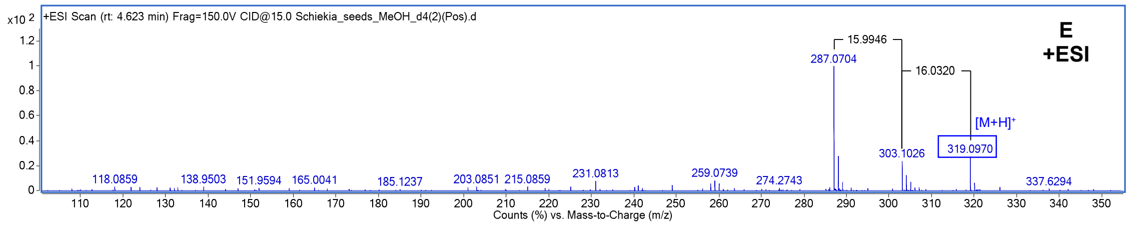

Figure S12. Mass spectrum of peak E (4.62 min) in positive ESI mode.

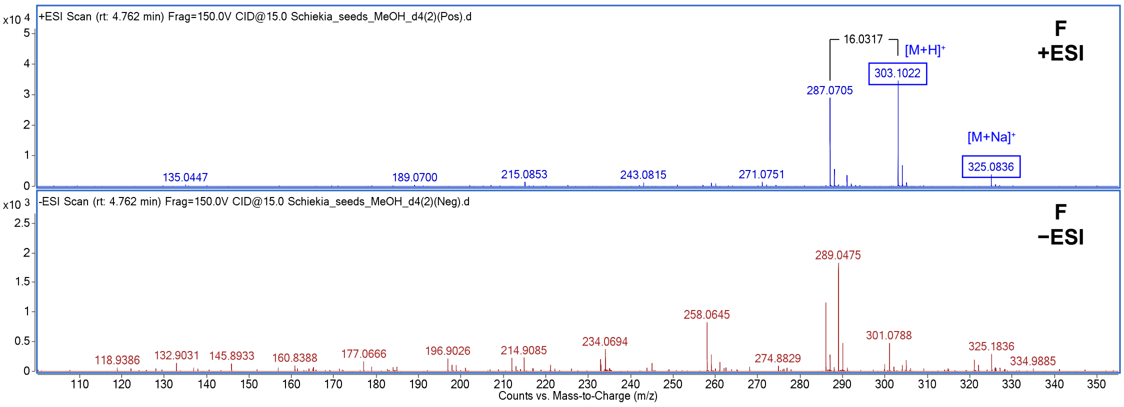

Figure S13. Mass spectra of peak F (4.75 min) in positive and negative ESI modes.

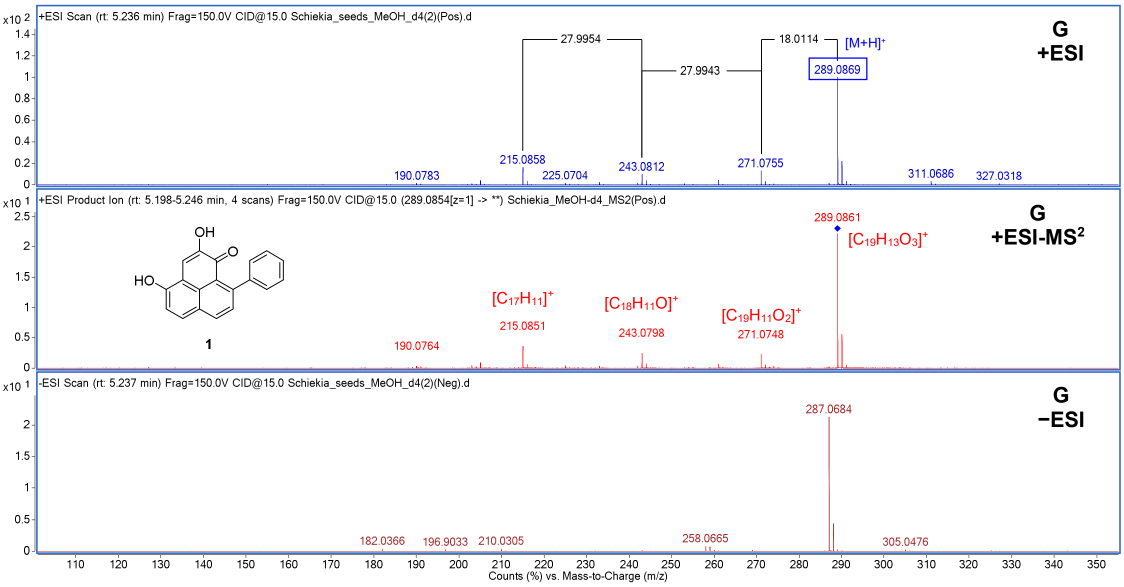

Figure S14. Mass spectra of peak G (5.23 min), compound **1**, in positive and negative ESI modes. MS^2 spectrum of precursor ion m/z 289.0861 with suggested molecular formulas for main fragments.

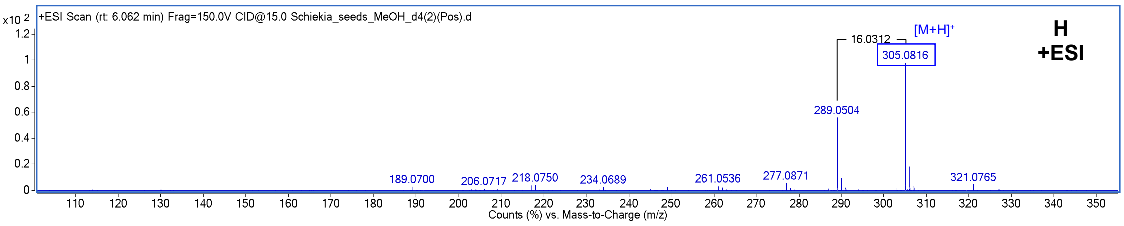

Figure S15. Mass spectrum of peak H (6.06 min) in positive ESI mode.

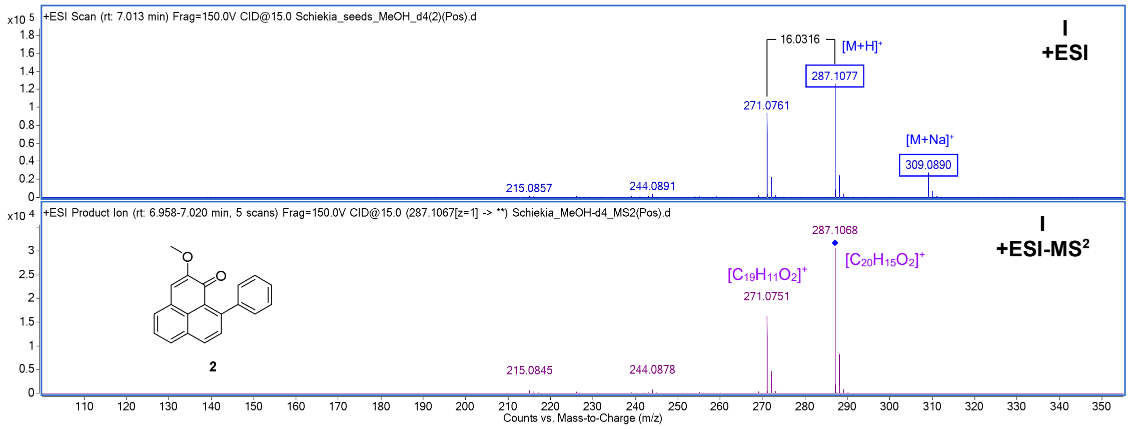

Figure S16. Mass spectra of peak I (7.00 min), compound **2**, in positive and negative ESI modes. MS[2] spectrum of precursor ion m/z 287.1068 with suggested molecular formulas for main fragments.

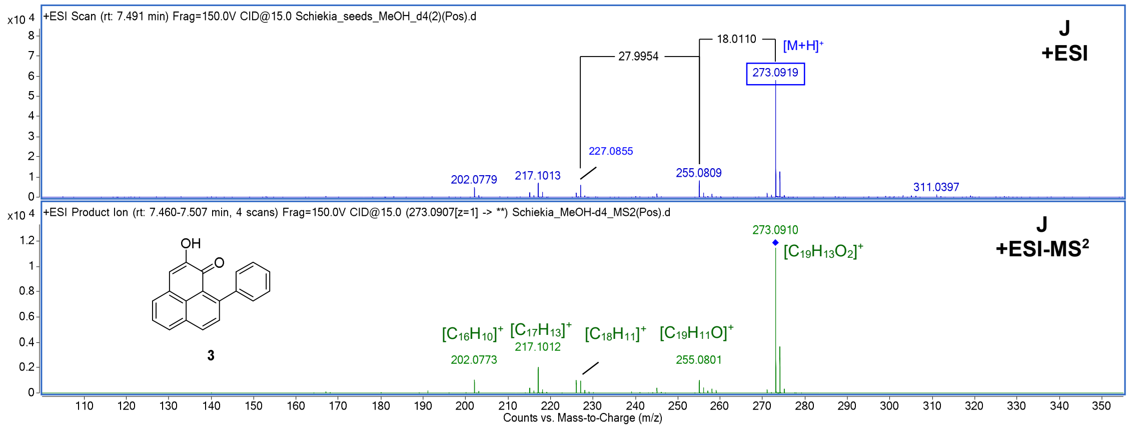

Figure S17. Mass spectra of peak J (7.49 min), compound **3**, in positive and negative ESI modes. MS[2] spectrum of precursor ion m/z 289.0861 with suggested molecular formulas for main fragments.
